# Genome-wide analysis of the cotton COBRA-like gene family and functional characterization of *GhCOBL22* in relation to drought tolerance

**DOI:** 10.1186/s12870-024-05965-x

**Published:** 2024-12-23

**Authors:** Wan-wan Fu, Zi-Yu Wang, Yun-Hao Liusui, Xin Zhang, Ai-Xia Han, Xing-Yue Zhong, Jing-Bo Zhang, Yan-Jun Guo

**Affiliations:** Xinjiang Key Laboratory of Special Species Conservation and Regulatory Biology, College of Life Science, Xinjiang Normal University, Urumqi, 830017 China

**Keywords:** COBL, Cotton, Drought stress, Biological function

## Abstract

**Background:**

The COBRA-like (COBL) gene family is a crucial glycosylphosphatidylinositol (GPI)-anchored proteins that participate in various biological processes in plants by regulating the arrangement of cell wall microfibrils. While the functions of *COBL* genes have been analyzed in several plant species, their roles in cotton’s response to abiotic stress remain unexplored.

**Results:**

This study identified and characterized the COBL gene family in *Gossypium hirsutum*, revealing a total of 39 COBL family members classified into five subgroups. Transcriptome analysis indicated that the transcription levels of several *GhCOBL* genes were upregulated following PEG treatment, with *GhCOBL22* being significantly induced. Further silencing of the *GhCOBL22* gene through virus-induced gene silencing (VIGS) technology demonstrated that this gene’s silencing reduced cotton’s drought stress tolerance. Under drought stress conditions, the activities of superoxide dismutase (SOD), peroxidase (POD), and catalase (CAT) enzymes, along with proline (PRO) content, were lower in *GhCOBL22*-silenced plants compared to control plants, while the accumulation of malondialdehyde (MDA) was significantly higher. Moreover, silencing the *GhCOBL22* gene also led to reductions in the levels of cellulose, hemicellulose, and lignin content in cotton leaves.

**Conclusion:**

A systematic survey of gene structure, motif composition, and evolutionary relationships of the *COBL* gene family was conducted in *Gossypium hirsutum*. Subsequent expression and functional studies indicated that *GhCOBL22* plays a significant role in cotton’s drought tolerance. These findings enhance our understanding of the biological functions of the COBL family and highlight the critical role of the *GhCOBL22* gene in cotton’s response to drought stress.

**Supplementary Information:**

The online version contains supplementary material available at 10.1186/s12870-024-05965-x.

## Introduction

Cotton is a significant economic crop and a strategic resource. Cotton fibers are vital raw materials for the textile industry, while its seeds provide a source of oil for the food industry. Currently, cotton is predominantly cultivated in arid and semi-arid regions, where its growth and development are severely threatened by drought. Drought substantially impacts both the yield and quality of cotton, resulting in considerable economic losses for cotton farmers and associated industries [1]. Some studies suggest that among all types of abiotic stresses affecting cotton, drought stress represents the most critical threat throughout its entire growth cycle, surpassing the cumulative effects of other abiotic stresses [2]. Consequently, breeding cotton varieties with improved drought tolerance is essential for sustainable cotton production.

The plant cell wall is a crucial component of the outer layer of plant cells, providing structural support and protection, while also determining cell shape and expansion. It acts as a natural barrier against adverse conditions and pathogens [3]. Plant cell walls are categorized into primary cell wall (PCW) and secondary cell wall (SCW). The primary cell wall is a thin layer of polysaccharides, including cellulose, hemicellulose (mainly xyloglucan), and pectin. It is permeable to small molecules and exhibits flexibility and extensibility, which are essential for normal cell growth. SCW forms after the cessation of cell growth and accumulates on the inner side of the primary wall. It is primarily composed of cellulose, with smaller amounts of hemicellulose (mainly xylan) and lignin. This secondary wall is abundant in vascular tissue tubular cells and fiber cells, providing the mechanical support necessary for upright growth. The hydrophobic lignin reinforces the cell wall structure of vascular tissue to resist negative pressure, facilitating efficient and continuous water transport within the plant. Research has demonstrated that alterations in the composition (e.g., cellulose, hemicellulose, pectin, and lignin), modifications (e.g., glycosylation and acetylation), and structure (e.g., microfibril arrangement) of the plant cell wall occur in response to biotic and abiotic stress, enhancing the plant’s resistance [4]. For instance, the UDP-galactose/glucose epimerase OsUGE3 in rice enhances biomass production and salt stress tolerance by promoting the accumulation of cell wall polysaccharides [5]. Cellulose synthase-like (Csl) genes are implicated in the synthesis of cell wall polysaccharides; overexpression of the *DcCslG3b* gene enhances transgenic tobacco’s drought stress tolerance [6, 7]. The Csl gene *OsCSLD4* positively regulates rice’s response to salt and osmotic stress by increasing abscisic acid biosynthesis [8]. Conversely, *OsBURP16* overexpression reduces the tolerance of rice to abiotic stress, as further analysis revealed that *OsBURP16* overexpression leads to pectin degradation, affecting cell wall integrity and transpiration rates [9]. Moreover, *OsNAC5* promotes lignin accumulation in roots by activating the expression of CINNAMOYL-CoA REDUCTASE 10, thereby enhancing drought resistance in rice [10]. *CmCAD2* and *CmCAD3* are responsible for synthesizing syringyl lignin and guaiacyl lignin monomers, which are essential for watermelon drought tolerance [11]. The pectin methylesterase gene *PtoPME35* positively regulates the plant’s response to drought, and its overexpression alters the methylesterification level of pectin [12]. Finally, the photo-sensitive leaf rolling 1 (*psl1*) gene in mutant rice plants enhances drought resistance, resulting in significant changes to the composition and structure of the cell wall in the mutant [13].

The COBL gene family encodes putative GPI-anchored proteins that regulate cell expansion and cellulose deposition in plants by influencing the orientation of cell wall microfibrils [14]. In several plant species, the *COBL* gene family has been systematically identified and analyzed. In the poplar genome, a total of 14 COBL genes have been identified. Co-expression network analysis indicates that *PtrCOBL2* and *PtrCOBL3* are associated with the response of poplar plants to abiotic stress [15]. In wheat, five COBL genes have been identified, and qPCR analysis revealed that *TaCOBL* genes respond to drought stress [16]. Zhao et al. (2022) identified 87 COBL genes across six *Rosaceae* species, including *PbCOBL12* and *PbCOBL13*, which are predominantly expressed during SCW formation [17]. DROT1 encodes a COBL protein, and research has shown that DROT1 can enhance cellulose content and maintain cellulose crystallinity, thereby modulating cell wall structure and improving drought resistance in rice [18]. In *Arabidopsis*, overexpression of *PtCOBL12* from *Pinus tabuliform* promotes plant growth, enhances cellulose content and relative crystallinity, and improves plant growth under drought stress conditions [19]. These findings indicate that COBL genes are involved in plant responses to abiotic stresses. Previous studies on the *GhCOBL* gene family have identified a total of 33 COBL genes within the cotton genome. Notably, *GhCOBL9* and *GhCOBL13* may play significant roles in cotton fiber development [20]. In addition, most existing research has primarily focused on the expression patterns of *COBL* genes under abiotic stress conditions, without thoroughly investigating their functional roles in stress responses. This project aimed to use the latest genomic data to identify *GhCOBL* genes and analyze their evolutionary relationships and the distribution of promoter *cis*-acting elements of this gene family. Screening focus on *COBL* gene family members implicated in cotton’s drought stress defense, using transcriptome data from PEG-treated conditions, followed by real-time quantitative PCR (qPCR) validation. Subsequently, we utilized VIGS technology to investigate the roles of *GhCOBL* gene family members in cotton’s drought stress response. This research will provide a foundation for further studies on the *COBL* gene family’s roles and mechanisms in cotton’s drought response.

## Results

### Identification and physicochemical property analysis of the COBL gene family in *Gossypium hirsutum*

A total of 39 COBL genes have been identified in the cotton genome. Based on their chromosomal positions, these genes have been designated as *GhCOBL1* to *GhCOBL39*. An analysis of the physicochemical properties of the *GhCOBL* gene family revealed significant variations in the lengths of their protein sequences, with the number of amino acids ranging from 109 to 667, with an average of 469.69 amino acids. The gene *GhCOBL36* has the fewest amino acids, consisting of only 109, while *GhCOBL2* and *GhCOBL21* each contain the highest number, with 667 amino acids. The isoelectric points of the GhCOBL proteins range from 4.55 to 9.26, and their molecular weights vary from 12,774.55 to 74,544.17 Da. Predictions regarding subcellular localization indicate that 14 *GhCOBL* genes are situated in the plasma membrane, 7 in the vacuole, 7 in the nucleus, 2 in the extracellular matrix, 2 in the cytoplasm, 1 in the golgi apparatus, 1 in the chloroplast and 3 in the endoplasmic reticulum (Table S1).

### Phylogenetic analysis of the *COBL* gene family

**Fig. 1:**
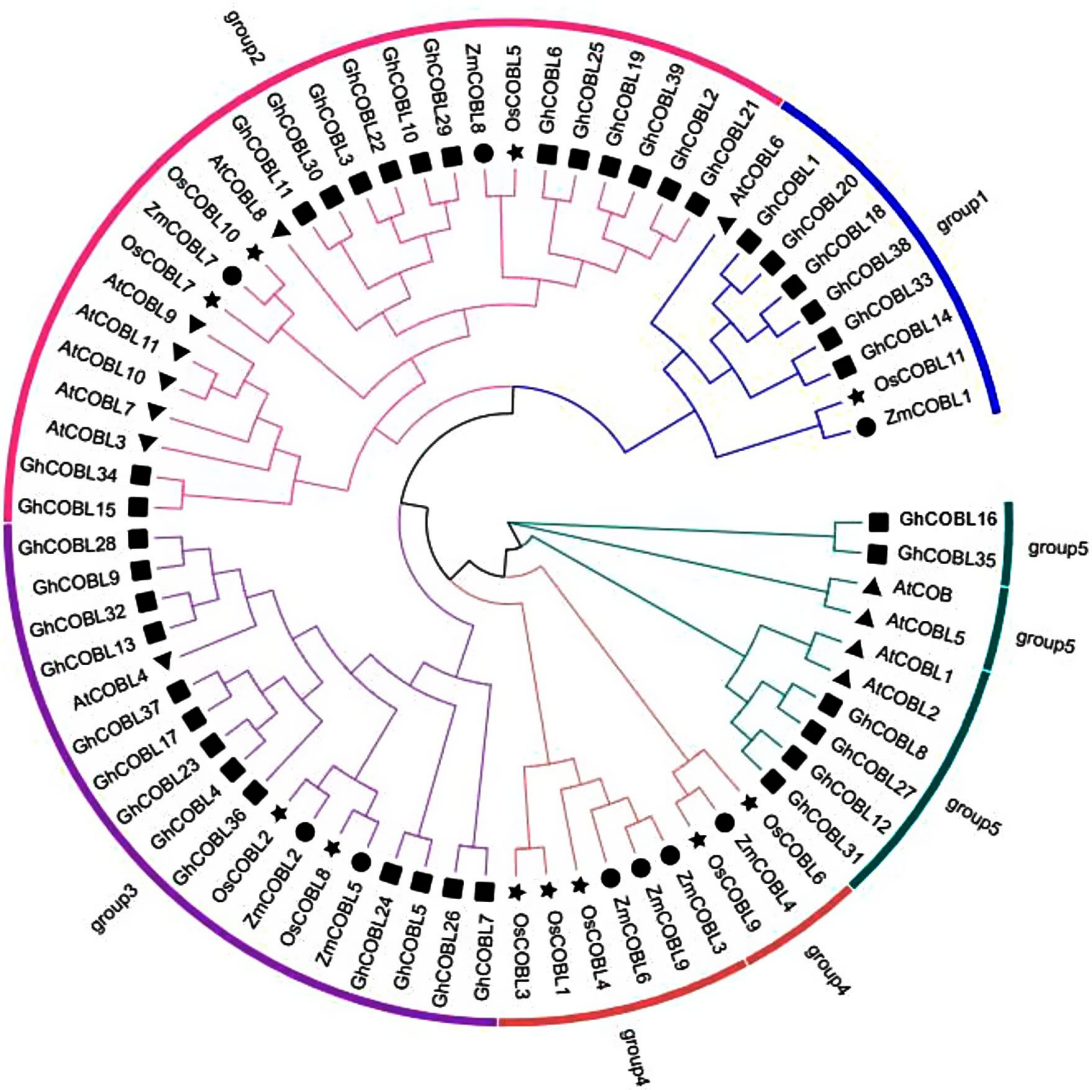
Phylogenetic analysis of the *COBL* gene family. The *GhCOBL* gene family is divided into five groups, with different colors representing different groups and different shapes representing different species

To elucidate the evolutionary relationships of the *COBL* gene family in cotton, we conducted a multiple sequence alignment using the protein sequences of 39 *GhCOBL* genes, 12 *Arabidopsis COBL* genes, 10 rice *COBL* genes, and 9 maize *COBL* genes, followed by the construction of a phylogenetic tree. The results of this analysis indicate that the *COBL* gene family can be divided into five distinct subgroups. The distribution of *GhCOBL* genes among these subgroups is uneven, with the second subgroup containing the largest number of *GhCOBL* gene members, totaling 14. The third subgroup comprises 13 *GhCOBL* genes, while the fifth and first subgroup includes 6 *GhCOBL* genes. Notably, no *GhCOBL* genes are found in group 4 (Fig. 1). The results suggest that the *GhCOBL* gene family in cotton has experienced functional differentiation throughout the course of evolution.

### Analysis of protein conserved motifs and gene structure of the GhCOBL family members in *Gossypium hirsutum*

**Fig. 2:**
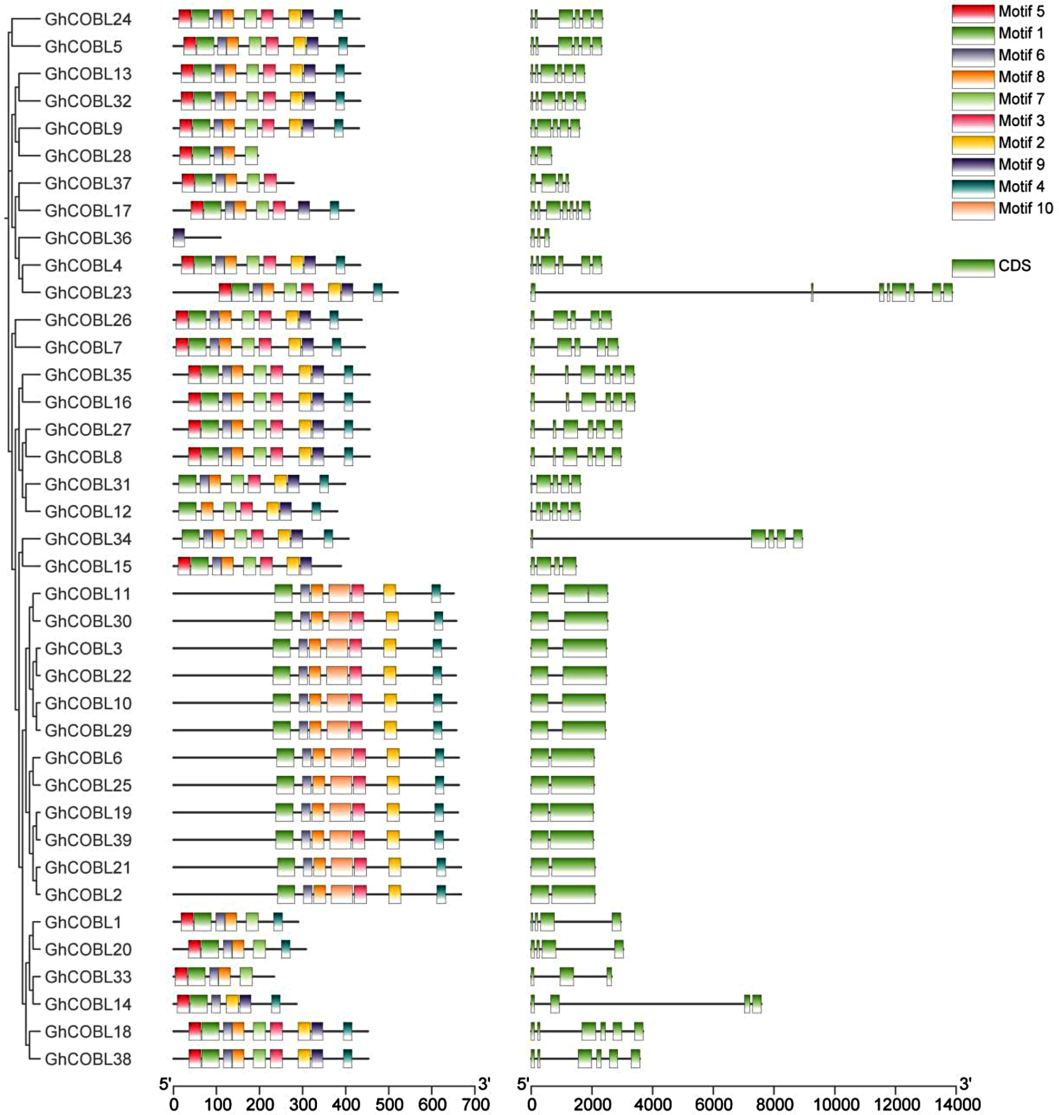
Analysis of the conserved protein motifs and gene structure of the *GhCOBL* gene family. The far left is the evolutionary analysis of GhCOBL proteins; the middle is the conserved motifs of GhCOBL proteins, with different colors representing different conserved motifs; and the right is the gene structure analysis, with green representing the CDS and black lines representing the intron

The results of the conserved motif analysis of the GhCOBL protein family indicate that closely related members exhibit a high degree of similarity in their motif composition. Notably, all GhCOBL proteins, with the exception of GhCOBL36, contain Motif 1, which suggests that Motif 1 is conserved among cotton GhCOBL proteins. Additionally, most cotton GhCOBL proteins also include Motif 2, Motif 4, and Motif 8, indicating that these three motifs are conserved features as well. Although the motif composition of the majority of GhCOBL proteins is generally similar, GhCOBL36 is missing certain motifs that are present in other GhCOBL members. Structural analysis of the *GhCOBL* genes reveals that the number of exons varies from 2 to 7, with closely related *GhCOBL* genes displaying similarities in their gene structures (Fig. 2).

### Analysis of *cis*-acting elements in the promoters of *GhCOBL* genes

**Fig. 3:**
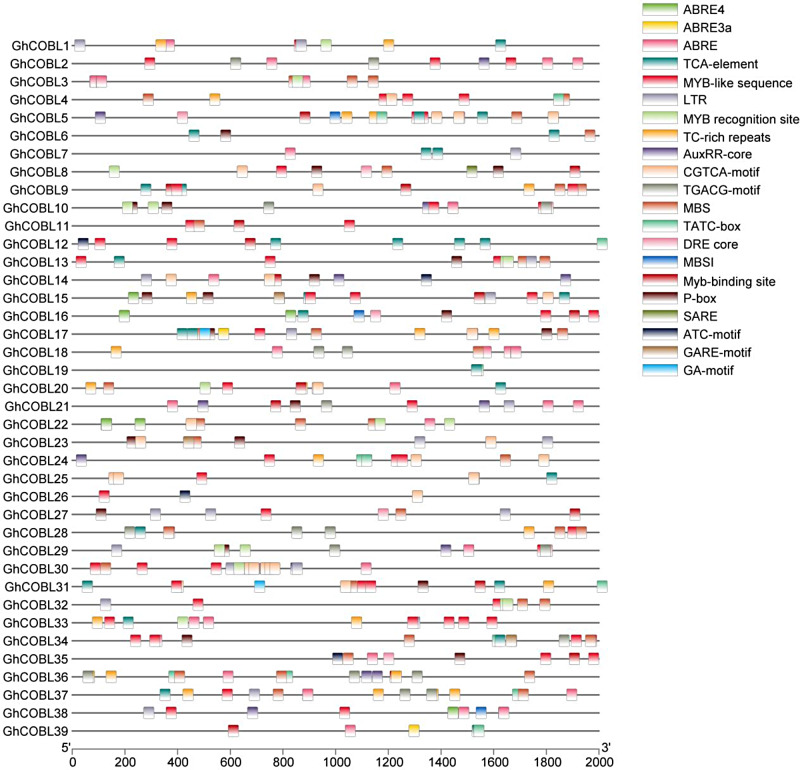
Analysis of *cis*-regulatory elements on *GhCOBL* gene promoters. Different colors represent different *cis*-acting elements

To further investigate the biological processes involving *GhCOBL* genes, we extracted a 2,000 bp sequence upstream of the start codon of *GhCOBL* genes to serve as promoter sequences and conducted a *cis*-acting element analysis. The results revealed that the promoter regions of COBL genes are enriched with elements associated with responses to abiotic stress, such as ABA-responsive elements (ABRE4, ABRE, ABRE3a), as well as drought stress response-related *cis*-acting elements like DRE core, MBS, and MBSI, which are widely distributed in the promoters of *GhCOBL* genes. This suggests that *GhCOBL* genes may play a significant role in cotton’s response to abiotic stress. Additionally, several *cis*-acting elements related to transcription factor regulation were identified; the majority of *GhCOBL* gene promoters contain binding sites for MYB transcription factors, indicating that the expression of *GhCOBL* genes may be regulated by these factors (Fig. 3).

### Expression analysis of the *GhCOBL* gene family in different tissues

**Fig. 4:**
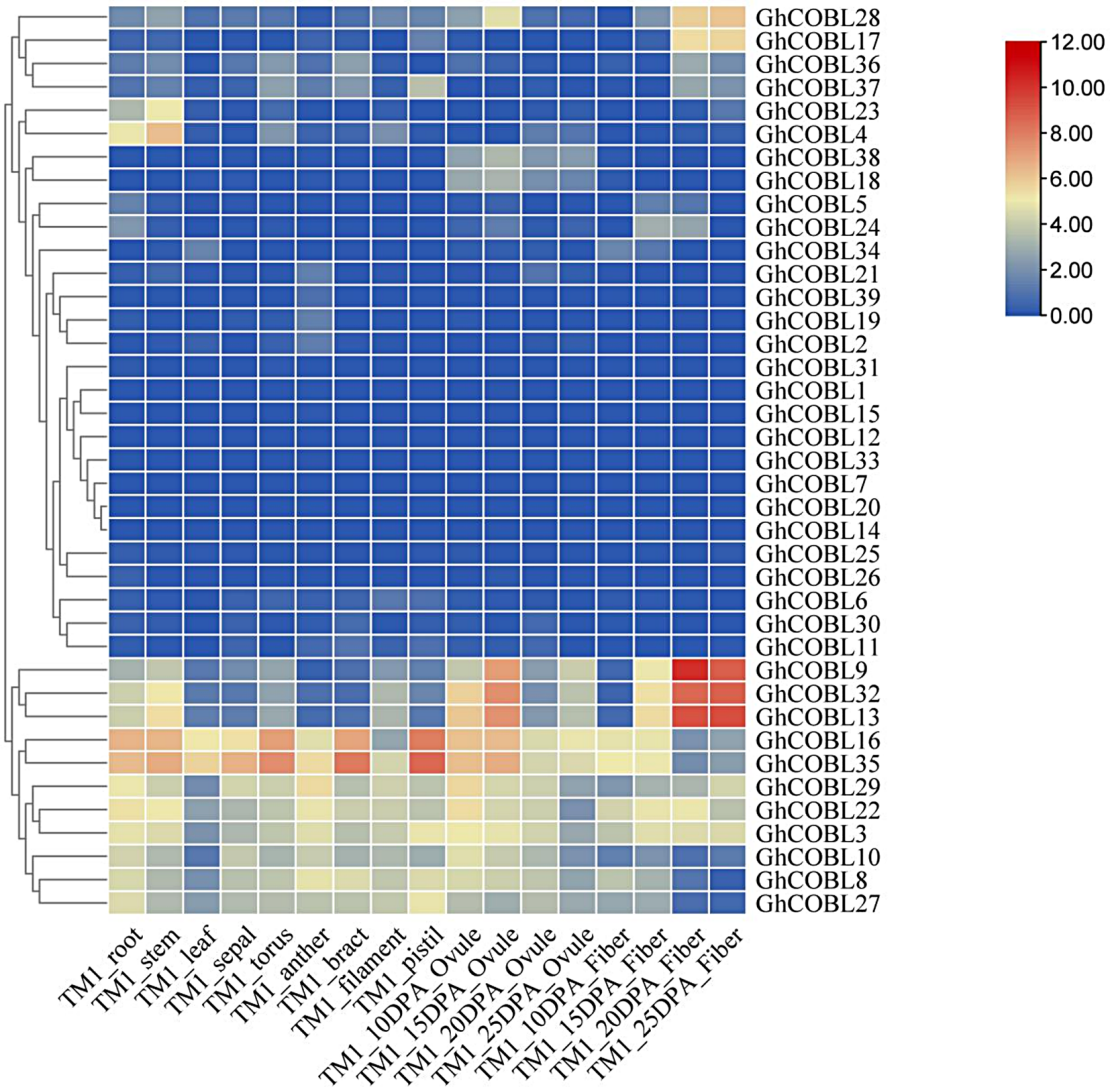
Analysis of expression patterns of *GhCOBL* family genes in cotton tissues. The gradient from blue to red indicates the expression levels of *GhCOBL* genes in different tissues of cotton, with colors closer to red representing higher expression levels

To investigate the involvement of *GhCOBL* genes in the growth and development of cotton, we utilized transcriptome data from various tissues of *Gossypium hirsutum*, including root, stem, leaf, sepal, torus, anther, bract, filament, pistil, ovule, and fiber. We conducted a detailed analysis of the tissue-specific expression patterns of *GhCOBL* genes, with the developmental stages of cotton ovules and fibers described in terms of days post-anthesis (DPA). The data analysis revealed that *GhCOBL* genes are expressed in all tissues, exhibiting varying expression levels across different tissues and developmental stages, indicating their complex biological functions throughout the cotton lifecycle. Several *GhCOBL* genes, including *GhCOBL1*, *GhCOBL7*, *GhCOBL12*, *GhCOBL14*, *GhCOBL15*, *GhCOBL20*, *GhCOBL25*, *GhCOBL26*, *GhCOBL31*, and *GhCOBL33*, show minimal expression in multiple cotton tissues. *GhCOBL28*, *GhCOBL17*, *GhCOBL9*, *GhCOBL32*, and *GhCOBL13* are expressed in fiber tissues, with higher expression levels observed in fibers at 20 DPA and 25 DPA, suggesting their potential roles in fiber development. *GhCOBL16* and *GhCOBL35* are expressed in several tissues of cotton. They exhibit the high expression levels in root, stem, leaf, sepal, torus, anther, bract, pistil, 10 DPA ovule, 15 DPA ovule (Fig. 4). These expression patterns indicate that these *GhCOBL* genes play roles in plant growth and development.

### Expression analysis of the *GhCOBL* gene family under PEG and drought treatment conditions

**Fig. 5:**
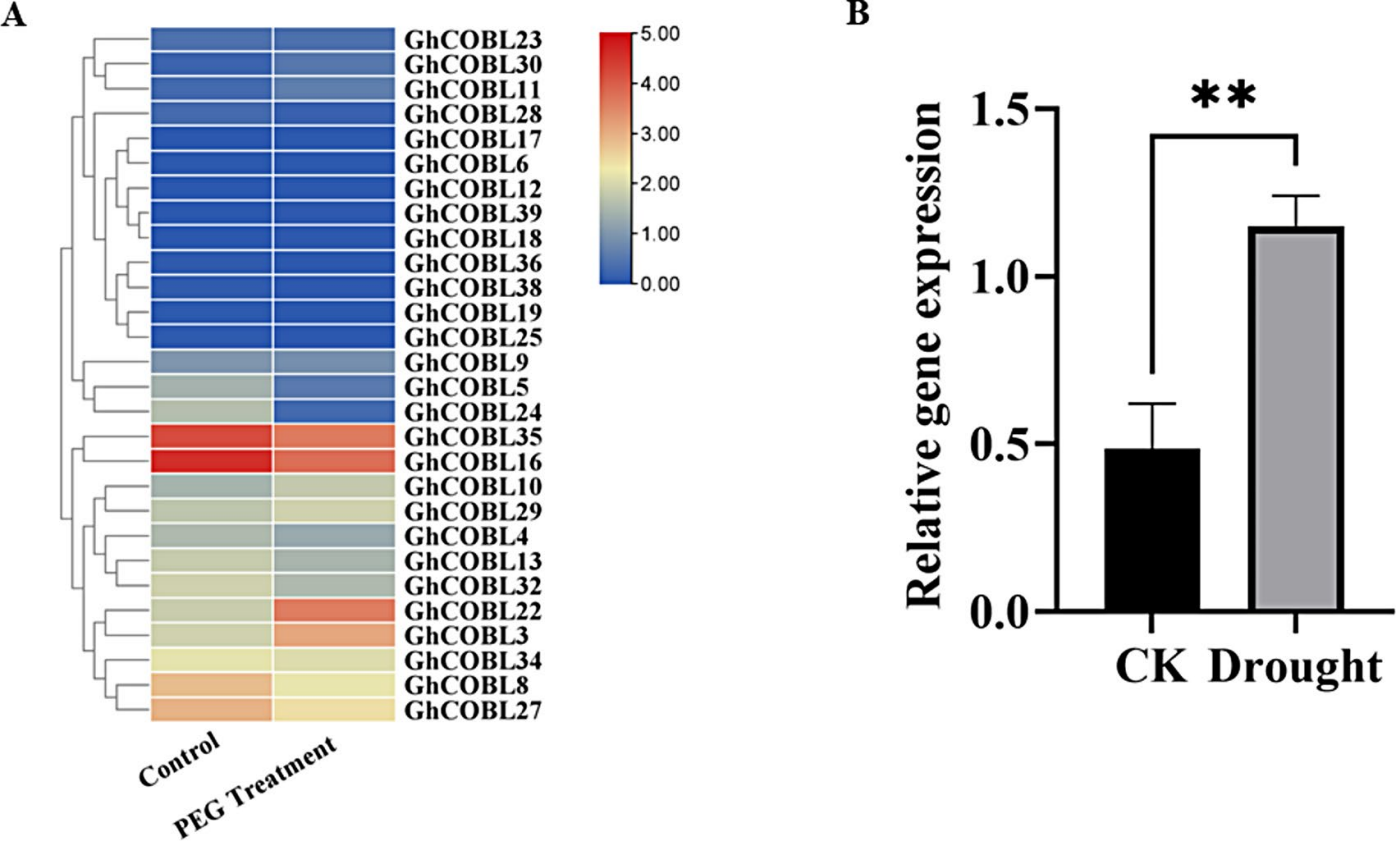
Expression pattern of *GhCOBL* genes under treatment conditions. (**A**) The expression analysis of *GhCOBL* gene family under PEG treatment. (**B**) The expression of *GhCOBL22* under drought stress. The asterisks indicate significant differences according to student’s *t*-test. *, *p* < 0.05; **, *p* < 0.01

To investigate the potential role of *GhCOBL* gene family members in drought stress, we examined the expression profiles of *GhCOBL* gene family members under PEG treatment conditions using transcriptome data. The data showed that the expression levels of some *GhCOBL* genes did not change significantly before and after PEG treatment. In contrast, the expression of *GhCOBL22* and *GhCOBL3* changed significantly under PEG treatment conditions. PEG treatment resulted in a significant up-regulation of *GhCOBL22* expression, suggesting that the *GhCOBL22* gene may be associated with the response of cotton to drought (Fig. 5A).

To further determine whether *GhCOBL22* is involved in responding to drought stress in cotton, we assessed the expression of *GhCOBL22* under normal watering and drought-treated conditions using qPCR. QPCR results indicated that the expression level of the *GhCOBL22* gene was elevated under drought stress conditions compared with the normal watering treatment group (Fig. 5B), a result that provides additional evidence that the *GhCOBL22* gene is involved in the response of cotton plants to drought stress.

Since there are some ABA-responsive elements on the promoters of *GhCOBL* genes (Fig. 3), we wondered whether *GhCOBL* genes could response to ABA. The expression of several *GhCOBL* genes induced by PEG were examined under ABA treatment. The result showed that *GhCOBL3* was up-regulated after treatment for 3 h, *GhCOBL29* had the highest expression at 12 h, and *GhCOBL10* and *GhCOBL22* showed up-regulation at 6 h, 12 h and 24 h. However the expression of *GhCOBL30* showed a down-regulation trend (Fig. S1).

### Functional validation of the *GhCOBL22* gene in the response of cotton to drought stress

**Fig. 6:**
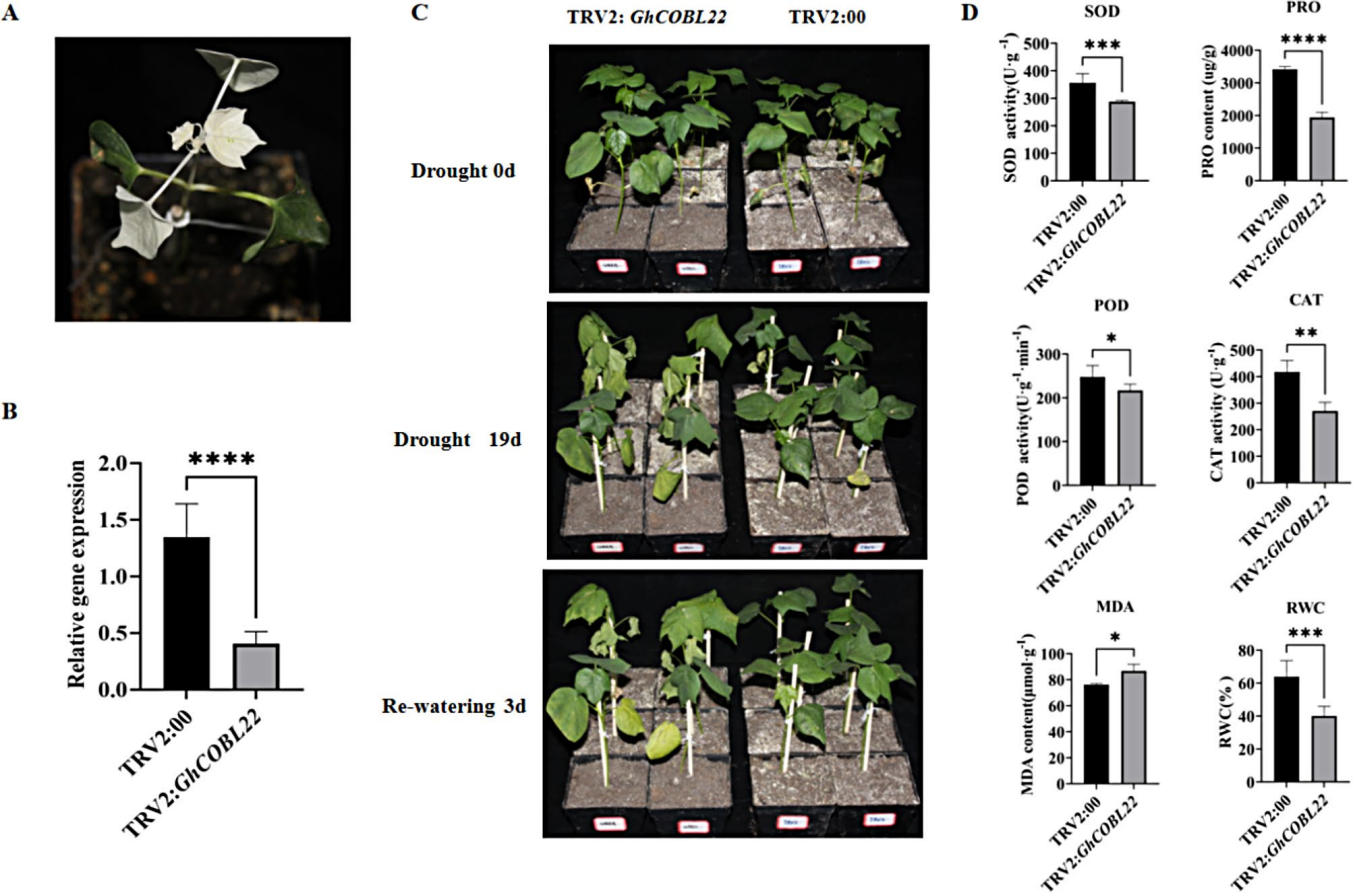
Silencing *GhCOBL22* reduces cotton’s tolerance to drought stress. **(A**) Positive control plants. (**B**) qPCR analysis of *GhCOBL22* expression in control plants (TRV2: 00) and VIGS plants (TRV2: *GhCOBL22*). The cotton *GhHis3* gene was used as the reference gene, and the error bars represent the standard deviation calculated from three independent experiments. (**C**) Phenotypes of TRV2: 00 and TRV2: *GhCOBL22* plants under drought stress. (**D**) Physiological indicators of TRV2: 00 and TRV2: *GhCOBL22* plants. The asterisks indicate significant differences according to student’s *t*-test. *, *p* < 0.05; **, *p* < 0.01; ***, *p* < 0.001; ****, *p* < 0.0001

To further investigate the role of the *GhCOBL22* gene in the response of cotton to drought stress, we utilized VIGS technology to silence this gene. Following the observation of changes in the positive plants, it was confirmed that the target gene had been effectively silenced (Fig. 6A). We measured the expression levels of *GhCOBL22* in TRV2:00 and TRV2:*GhCOBL22* plants to verify whether the expression of the *GhCOBL22* gene was significantly suppressed. The results indicated that the expression level of the *GhCOBL22* gene in TRV2:*GhCOBL22* plants was significantly lower than that in TRV2:00 plants, confirming the effective silencing of the *GhCOBL22* gene (Fig. 6B).

Subsequently, we subjected 3-week-old TRV2:00 and TRV2:*GhCOBL22* plants to drought treatment. At day 0 of drought treatment, there were no apparent phenotypic differences between the TRV2:00 and TRV2:*GhCOBL22* plants. However, after 19 days of drought treatment, the growth conditions of the TRV2:00 plants were significantly better than those of the TRV2:*GhCOBL22* plants, which exhibited more severe wilting compared to the TRV2:00 plants (Fig. 6C). We further examined the physiological indicators of the cotton leaves under drought treatment. The results indicated that under drought stress conditions, the content of PRO and relative water content (RWC), as well as the activities of the SOD, POD and CAT enzymes in TRV2:*GhCOBL22* cotton plants, were significantly lower than those in TRV2:00 plants. Drought stress resulted in a significant increase in MDA accumulation in TRV2:*GhCOBL22* cotton plants compared to TRV2:00 plants (Fig. 6D). The correlation between physiological parameter and transcription level was assessed using Pearson correlation coefficient. The results showed that the expression level of *GhCOBL22* was positively correlated with the activities of SOD, POD and CAT enzymes, as well as the content of PRO and relative water content (RWC) in TRV2:00 and TRV2:*GhCOBL22* plants under drought stress. And the expression level of *GhCOBL22* was negatively corelated with MDA content (Fig. S2). Collectively, the results of the phenotypic analysis and physiological measurements indicate that silencing the *GhCOBL22* gene reduces the drought tolerance of cotton.

### Inhibiting the expression of the *GhCOBL22* gene affects the content of cell wall components

**Fig. 7:**
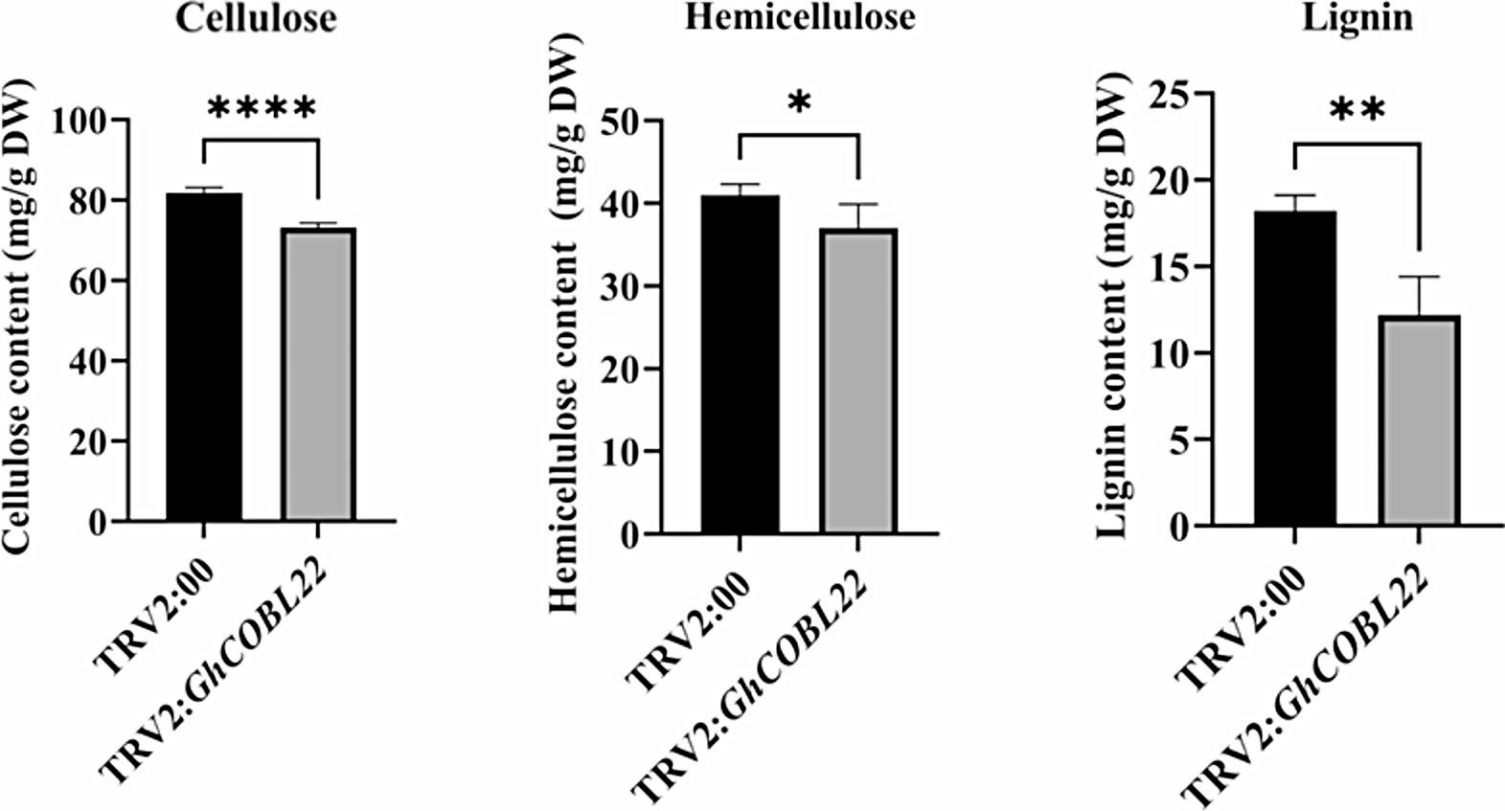
Silencing *GhCOBL22* affects cell wall properties under drought stress conditions. DW represents the dry weight of the sample. The asterisks indicate significant differences according to student’s *t*-test. *, *p* < 0.05; **, *p* < 0.01; ***, *p* < 0.001; ****, *p* < 0.0001

To verify whether *GhCOBL22* affects cell wall component levels under drought conditions, we measured the levels of cellulose, hemicellulose, and lignin in the leaf tissues of both the control and silenced cotton plants. Under drought conditions, the cellulose, hemicellulose, and lignin contents in the control plants were significantly higher than those in the silenced plants (Fig. 7). Correlation analysis showed that the expression level of *GhCOBL22* was positively correlated with cellulose, hemicellulose and lignin content (Fig. S3). These results indicate that silencing *GhCOBL22* affects the accumulation of cellulose, hemicellulose, and lignin in drought conditions.

## Discussion

The COBL family in plants plays a crucial role in growth, development, and responses to biotic and abiotic stresses [21]. Previously, COBL genes have been identified in various plants. For instance, diploid plants like *Arabidopsis* [22], maize [23], and soybean [24] exhibit 12, 8, and 24 COBL genes, respectively. In this study, we discovered that the cotton genome comprises 39 COBL genes, which is significantly higher than the number observed in diploid plants such as *Arabidopsis* and maize. This suggests that the increased number of COBL gene family members in cotton is likely due to genomic duplication events that occurred in tetraploid plants.

Genes that are closely related often have similar biological functions [25, 26]. In *Arabidopsis*, AtCOBL is involved in the directional growth of cellulose microfibrils and plays a crucial role in the highly specific amplification of plant morphology [27]. *GhCOBL8*, *GhCOBL12*, *GhCOBL27*, and *GhCOBL31* exhibit high homology with *AtCOBL*, suggesting their potential involvement in the directional orientation of cellulose microfibrils in cotton. *AtCOBL4* is essential for cellulose synthesis in the secondary wall and impacts the mechanical strength of plant tissues [28]. *GhCOBL9*, *GhCOBL13*, *GhCOBL28*, and *GhCOBL32* are homologous to *AtCOBL4* and may be involved in cellulose synthesis in the SCW.

Previous studies have shown that members of the COBL gene family play a role in plant responses to abiotic stress [16]. The expression of barley *HvCOBL3*, *HvCOBL4*, and *HvCOBL11* is induced by ABA treatment [29]. The transcription level of poplar *PtrCOBL10* increases under high temperature and salt stress conditions [15]. Analysis of *Liriodendron chinense* LcCOBL’s response to abiotic stress using RNA-seq revealed that *LcCOBL3* responds to both drought and heat stress [30]. Transcriptome data from cotton leaves subjected to PEG treatment showed a significant induction of multiple *GhCOBL* genes. Additionally, promoter sequences of *GhCOBL* genes contain several *cis*-acting elements related to drought stress response, indicating the involvement of the *GhCOBL* gene family in cotton’s drought stress response. This further demonstrates the association of the *COBL* gene family with plant responses to abiotic stress. In *Arabidopsis*, overexpression of *COBL9* significantly increases root hair elongation and enhances plant tolerance to salt stress [31]. Researchers discovered that DROT1 encodes a COBL protein that positively regulates drought resistance in rice [18]. In this study, we found that silencing the *GhCOBL22* gene weakened cotton’s ability to withstand drought stress, further underscoring the important role of the *COBL* gene family in plant responses to abiotic stress.

The SCW of plants is primarily composed of cellulose, hemicellulose, and lignin, providing mechanical support to the plant. Additionally, the structural components of the cell wall play crucial roles in the plant’s ability to withstand various abiotic stresses [32]. The DROT1 gene in rice encodes a COBL protein that enhances drought resistance by regulating cell wall structure and increasing cellulose content [18]. Studies have shown that overexpressing *MsMIOX2* in alfalfa promotes hemicellulose biosynthesis, increases cell wall thickness, and enhances alfalfa’s tolerance to salt stress [33]. In *Arabidopsis*, overexpression of *COBL9* promotes cellulose deposition in root hairs, significantly increasing root hair elongation and improving the plant’s tolerance to salt stress [31]. COBL7 and COBL8 in *Arabidopsis* regulate the mechanical properties of guard cell walls through direct interaction with cellulose, influencing the development and function of guard cell morphology, which further affects the plant’s sensitivity to drought stress [34]. Under drought stress, *PuEARLI1* overexpression increases lignin content, enhancing mechanical support, resulting in greater drought tolerance in poplar [35]. These studies suggest that altering the components or structure of the cell wall can modify a plant’s resistance to drought stress. In this study, we identified *GhCOBL22*, which is significantly upregulated under PEG treatment. Silencing *GhCOBL22* in cotton plants resulted in lower levels of cellulose, hemicellulose, and lignin compared to control plants, indicating that *GhCOBL22* participates in the plant’s response to drought stress by affecting the composition of the cotton cell wall. Our findings, consistent with previous research, demonstrate that the structural components of the cell wall are closely related to the plant’s response to abiotic stress.

When plants experience drought stress, the balance of their reactive oxygen species (ROS) metabolic system is disrupted, leading to the accumulation of ROS molecules such as superoxide (O^2-^), hydrogen peroxide (H_2_O_2_), and hydroxyl radicals (OH^-^). Excessive ROS accumulation intensifies lipid peroxidation, causing membrane damage [36]. The content of MDA is commonly used as an indicator of lipid peroxidation in cell membranes and can directly reflect the extent of ROS induced damage to plant cells [37]. Plants have developed a comprehensive ROS scavenging system to mitigate ROS accumulation caused by stress, with SOD, CAT, and POD being key components of this system [38]. Studies have found that the activities of SOD, CAT, and POD are closely related to plant drought resistance, as these enzyme activities significantly increase under drought stress [39, 40]. In this study, under drought stress conditions, the silenced cotton group exhibited lower activities of SOD, POD, and CAT compared to the control group, while MDA levels were higher. This indicates that the inhibition of *GhCOBL22* gene transcription weakened the cotton’s ability to scavenge ROS, resulting in more severe cellular damage and reduced tolerance to drought stress. PRO is a free osmotic regulator. When plant cells face adverse stresses, they accumulate such osmotic regulators to lower their cellular osmotic potential, preventing excessive water loss and thereby enhancing drought resistance [41]. In cotton plants with silenced *GhCOBL22*, the PRO content was lower than that of the control plants, suggesting that the downregulation of *GhCOBL22* expression affected PRO accumulation, leading to decreased water retention capacity and increased sensitivity to drought stress.

## Materials and methods

### Identification of *GhCOBL* gene family members

The genome sequence and protein sequence of *Gossypium hirsutum* (TM1_WHU) were downloaded from the CottonMD database (https://yanglab.hzau.edu.cn/CottonMD) [42]. Using AtCOBL protein sequences from *Arabidopsis thaliana* as probes, local BLASTP was employed to predict COBL gene family members in cotton, with an E-value threshold set to < *e*-10. The results predicted by BLASTP were subsequently submitted to the Pfam database (pfam.xfam.org/) to identify the COBL domain (PF 04833), thereby confirming the COBL gene family members within the *Gossypium hirsutum* genome. To analyze the physicochemical properties of the GhCOBL protein sequences, the ProtParam tool on the ExPASy website was utilized to gather information regarding molecular weight, isoelectric point, and the number of amino acids in the COBL proteins. The GhCOBL protein sequences were then submitted to the WoLF PSORT database (https://wolfpsort.hgc.jp/) to predict their subcellular localization.

### Phylogenetic analysis of the *GhCOBL* gene family

COBL family protein sequences from rice (*Oryza sativa*) and maize (*Zea mays*) were retrieved from the Phytozome database (https://phytozome-next.jgi.doe.gov/). Multiple sequence alignments of the COBL protein sequences were conducted using MEGA 11 software. Based on the alignment results, a phylogenetic tree was constructed using FastTree software with the neighbor-joining method, employing 1,000 bootstrap replications. The resulting phylogenetic tree was subsequently visualized using Evolview (https://evolgenius.info/evolview-v2/#login).

### Structural and conserved motif analysis of the *GhCOBL* genes

The protein sequences of the *GhCOBL* genes were analyzed using MEME Suite 5.1.0 (https://meme-suite.org/meme/tools/meme) to predict conserved motifs, with the number of motifs set to 20 and default parameters applied for all other settings. Subsequently, genomic and annotation files for cotton were retrieved from the CottonMD database, and these annotation files were imported into TBtools software to extract the structural information of the COBL genes.

### Analysis of *Cis*-acting elements in the promoters of *GhCOBL* gene family members

The 2,000-bp sequences upstream of the start codon (ATG) for members of the *GhCOBL* gene family were extracted as promoter sequences. These promoter sequences were submitted to PLANTCARE (http://bioinformatics.psb.ugent.be/webtools/plantcare/html/) for the identification and characterization of *cis*-acting elements. The information regarding *cis*-acting elements obtained from the COBL promoters was subsequently visualized using TBtools.

### Expression analysis of *GhCOBL* gene family members

Expression data for the *GhCOBL* gene family in various cotton tissues, as well as at different developmental stages of fibers and ovules, were obtained from CottonMD (https://yanglab.hzau.edu.cn/CottonMD/heatmap.1). The expression levels of the transcriptome data were standardized to log_2_-transformed TPM values. Transcriptome sequencing was performed on cotton leaves treated with 18% PEG6000 hydroponics for 4 h. The data were deposited in the OMIX, China National Center for Bioinformation / Beijing Institute of Genomics, Chinese Academy of Sciences (https://ngdc.cncb.ac.cn/omix: accession no. OMIX007375) (Database Resources of the National Genomics Data Center, China National Center for Bioinformation, 2024). The expression data of COBL genes was extracted from the transcriptome, and then a heatmap was generated using TBtools.

To investigate the response of *GhCOBL* to ABA, cotton seedlings with uniform growth were selected and leaves were treated with 100 µM of ABA solution and samples were collected at 0 h, 3 h, 6 h, 12 h and 24 h of treatment. Transcript levels of *GhCOBL* under ABA stress were analysed using qPCR-specific primers (Table S2). Each sample was analysed using 3 biological replicates and each biological replicate was analysed using 3 technical replicates. *GhHis3* was used as an internal reference gene [43] and the 2^-ΔΔCt^ method was used to calculate relative expression values.

### Plant material and growth conditions

The seeds of the *Gossypium hirsutum* variety Zhongmian 49 were provided by Xinjiang Zhongmian Seed Industry Co., Ltd. (Aksu City, Xinjiang, China). Cotton seeds were germinated on moist filter paper and then planted in pots containing a mixture of nutrient soil and vermiculite (in a 3:1 volume ratio). The soil in each pot was pre-weighed to ensure a consistent volume of soil. Cotton seedlings were cultured in a greenhouse at 28 °C with a photoperiod of 16 h of light and 8 h of darkness.

### RNA extraction and qPCR analysis

Total RNA was extracted from cotton leaves using a plant polysaccharide and polyphenol RNA extraction kit (FOREGENE, Chengdu, China), in accordance with the manufacturer’s instructions. The integrity of the extracted RNA was assessed via agarose gel electrophoresis, and the concentrations of the RNA samples were measured using a NanoDrop 2000 spectrophotometer (Thermo Fisher Scientific, Wilmington, DE, USA). The RNA was reverse transcribed into cDNA using the Master Premix (for qPCR) RT EasyTM II reverse transcription kit (FOREGENE, Chengdu, China). QPCR was conducted employing the SYBR Green I dye method with Real Time PCR EasyTM-SYBR Green I reagents (FOREGENE, Chengdu, China). The *GhHis3* gene served as the internal reference gene for the qPCR reactions, and the expression levels of the target genes were calculated using the 2^-ΔΔCt^ method. All experiments were performed with three biological replicates, and all primers utilized in this study are listed in Supplementary Table S2.

### VIGS experiment

The VIGS experiment employed the Tobacco Rattle Virus (TRV) dual expression vectors, TRV1 and TRV2. TRV1 carries the genes encoding RNA-dependent RNA polymerase, movement protein, and 16 kD protein, serving as an auxiliary viral vector within the VIGS system. TRV2 contains the capsid protein (CP) gene and multiple cloning sites (MCS), which facilitate the insertion of fragments for the silencing of target genes. The *GhCLA* gene, known for its high conservation and role in chloroplast development, displayed a distinct whitening phenotype upon silencing, thereby serving as a positive control for the VIGS experiment [44].

A specific 400-bp sequence of the *GhCOBL22* gene was selected as the target sequence, and primers were designed to amplify this sequence from a cotton cDNA library. The TRV2:*GhCOBL22* recombinant vector was constructed, and the TRV1, TRV2, and TRV2:*GhCOBL22* vectors were separately introduced into Agrobacterium GV3101. The Agrobacterium containing TRV1 was then mixed with Agrobacterium containing TRV2, TRV2:*GhCLA*, and TRV2:*GhCOBL22*, and subsequently injected into cotton cotyledons. Plants injected with the TRV1 and TRV2 strains served as controls and were designated TRV2:00, while those injected with TRV1 and TRV2:*GhCOBL22* were classified as the *GhCOBL22* gene-silenced lines, referred to as TRV2:*GhCOBL22*. The observation of a whitening phenotype in TRV2:*GhCLA* plants indicated successful silencing of the target gene. RNA was subsequently extracted from the leaves of TRV2:00 and TRV2:*GhCOBL22* plants 23 days after injection for qPCR analysis to assess whether the *GhCOBL22* gene had been effectively silenced. Cotton plants in which the *GhCOBL22* gene was successfully silenced were selected for drought treatment and further analysis.

### Drought treatment experiment

Consistent TRV2:00 and TRV2:*GhCOBL22* cotton plants were selected and cultured under identical conditions. Prior to the drought treatment, equal amounts of water were administered to the cotton plants, after which watering was ceased to facilitate natural drought conditions. Photographs were taken daily to document the phenotypic changes in the cotton plants.

### Measurement of physiological indicators and cell wall components

Leaves from TRV2:00 and TRV2:*GhCOBL22* cotton plants subjected to drought treatment for 20 days were collected, quickly frozen in liquid nitrogen, and ground into a powder. The activities of SOD, POD and CAT, the contents of PRO, MDA, cellulose, hemicellulose, and lignin were quantified using corresponding reagent kits (Grace, Suzhou, China) (http://www.geruisi-bio.com/pro). The brief procedures were as follow. Adopting the WST-8 method, SOD activity was calculate by measuring the absorbance at 450 nm using a spectrophotometer. Mix H₂O₂, guaiacol, enzyme extract and buffer solution, then incubate the reaction at 37 °C. Measure the absorbance of the reaction mixture at 470 nm using a spectrophotometer and calculate the POD enzyme activity. Mix H₂O₂, enzyme extract and buffer solution, then incubate the reaction at 37 °C. Add 1 M H₂SO₄ to stop the reaction. Measure the absorbance of the reaction mixture at 240 nm using a spectrophotometer to calculate CAT enzyme activity. PRO was extracted using sulfosalicylic acid, followed by heating and reaction with acidic ninhydrin. Measure the absorbance of the reaction mixture at 520 nm using a spectrophotometer to calculate PRO content. MDA condensed with thiobarbituric acid under heating conditions could generate a red product. The absorbance at 532 nm and 600 nm is measured and the difference between the absorbances is used to calculate the amount of MDA. To measure the RWC of leaves, the fresh weight (Wf) is recorded, followed by immersion in distilled water for 5–8 h to achieve saturation (saturated fresh weight, Wt). After drying for 5–8 h, the dry weight (Wd) is obtained. The RWC is calculated as: (Wf − Wd) / (Wt − Wd) × 100%.

To measure the content of cell wall components, the alcohol-insoluble residues (AIR) of the samples were extracted with 80% alcohol and treated with DMSO to remove starch. Starch-removed AIR was hydrolyzed by heating under acidic conditions. To measure cellulose content, take an appropriate amount of hydrolysed solution and add 98% H₂SO₄ to dehydrate glucose to form furfural compounds. The cellulose content was determined by quantifying the absorbance at 620 nm of the blue-green solution formed from the reaction between anthrone reagent and furfural compounds. Hemicellulose could hydrolyzed under acidic conditions to generate xylose, and the absorbance at 460 nm is measured to assess hemicellulose content. The acetylation method is employed to acetylate the phenolic hydroxyl groups in lignin, with lignin content calculated by measuring the absorbance at 280 nm.

## Conclusion

This study identified 39 *GhCOBL* genes in cotton, which are involved in developmental processes and abiotic stress responses. *GhCOBL22* was found to play a crucial role in drought resistance. Silencing *GhCOBL22* reduced drought tolerance, as evidenced by decreased enzyme activities, lower osmotic regulators, and altered cell wall components. These findings lay the groundwork for future genomics-assisted breeding to enhance cotton’s drought resistance.

## Electronic Supplementary Material

Below is the link to the electronic supplementary material.

Supplementary Material 1: Figure S1. *GhCOBL* gene expression under ABA treatment conditions. Figure S2. Analysis of the correlation between physiological parameters and transcription level using Pearson’s correlation coefficients. Figure S3. Analysis of the correlation between cell wall components and transcription level using Pearson’s correlation coefficients. Table S1. Characteristics of *GhCOBL* family genes in *Gossypium hirsutum*. Table S2. The primers used in this study.

## Data Availability

Data is provided within the manuscript or supplementary information files.
