# Supplementary material for: Genome-wide analysis of the cotton COBRA-like gene family and functional characterization of GhCOBL22 in relation to drought tolerance
Source: BMC Plant Biol. 2024 Dec 23;24:1242. doi: 10.1186/s12870-024-05965-x (PMC11668015; doi:10.1186/s12870-024-05965-x)
Supplement: Supplementary file 1 — Supplementary Material 1: Figure S1. GhCOBL gene expression under ABA treatment conditions. Figure S2. Analysis of the correlation between physiological parameters and transcription level using Pearson’s correlation coefficients. Figure S3. Analysis of the correlation between cell wall components and transcription level using Pearson’s correlation coefficients. Table S1. Characteristics of GhCOBL family genes in Gossypium hirsutum. Table S2. The primers used in this study. [file 12870_2024_5965_MOESM1_ESM.docx]

**Supplementary Information**

**
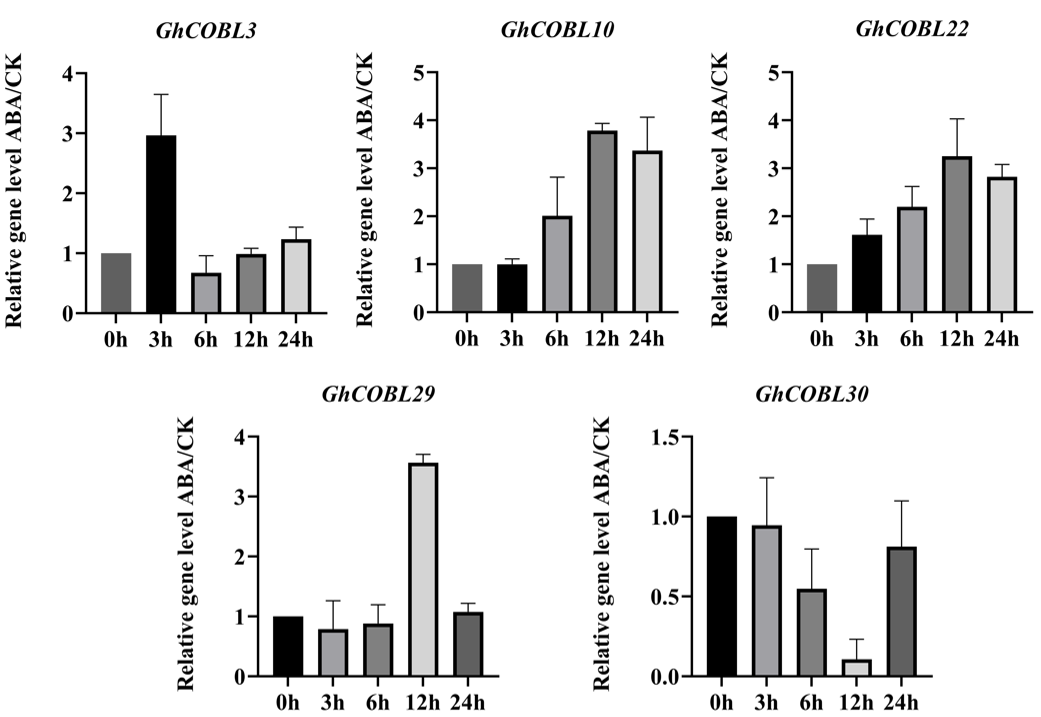
**

**Fig. S1 *GhCOBL* gene expression under ABA treatment conditions.**

The expression of several *GhCOBL* genes induced by PEG were examined under ABA treatment. The horizontal axis is the time of ABA treatment, and the vertical axis is the ratio of gene expression under ABA treatment conditions to that under CK conditions at the same time.


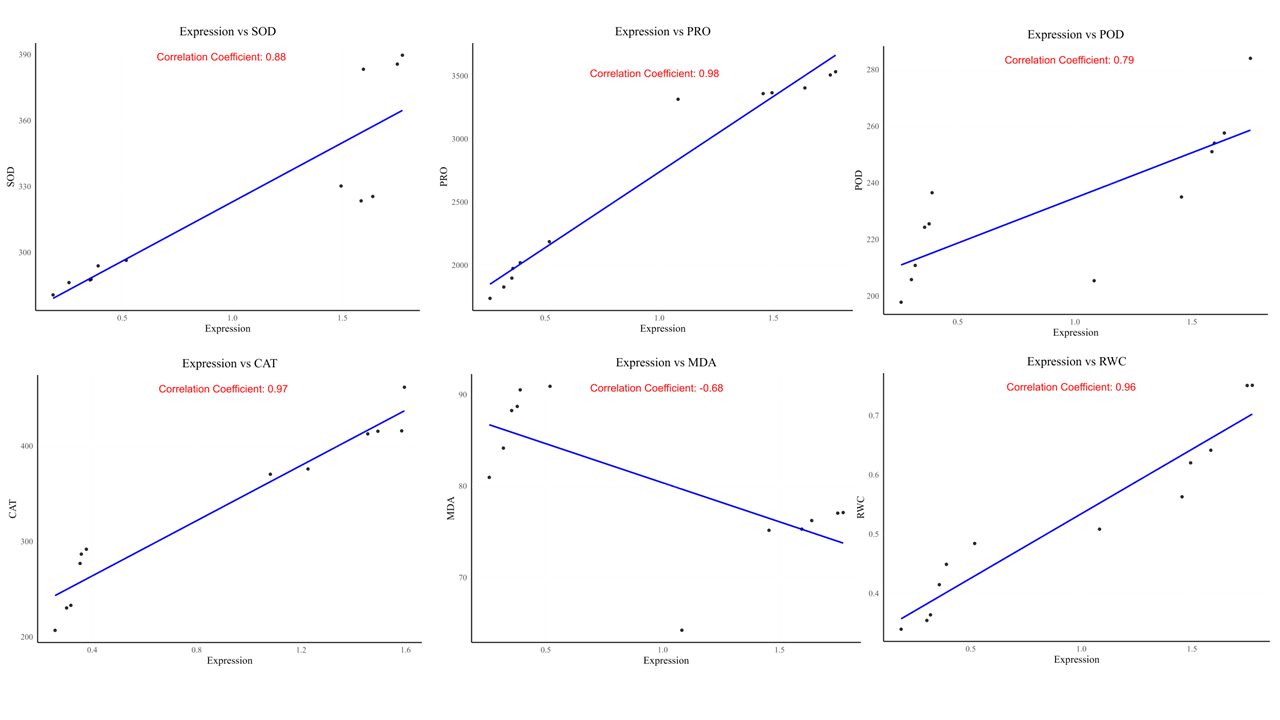


**Fig. S2 Analysis of the correlation between physiological parameters and transcription level using Pearson’s correlation coefficients.**

The Pearson function in the R language was utilized to compute the correlation coefficient between transcript levels and various physiological indicators.

**
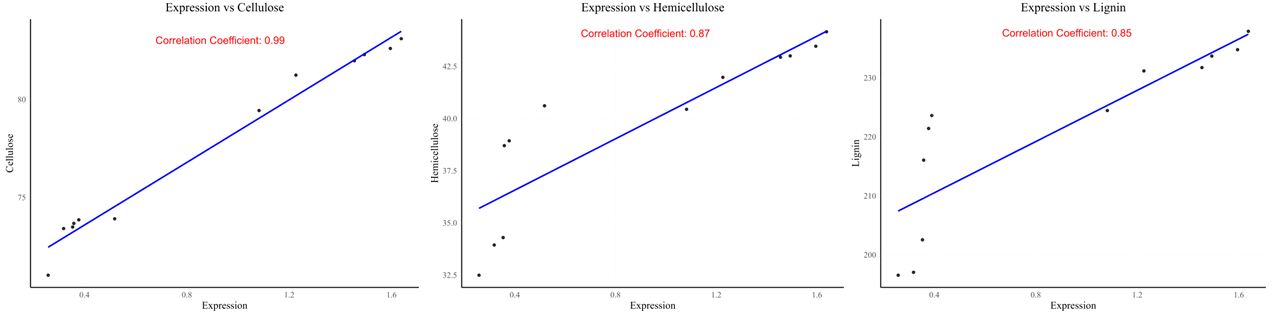
**

**Fig. S3 Analysis of the correlation between cell wall components and transcription level using Pearson’s correlation coefficients.**

The Pearson function in the R language was utilized to compute the correlation coefficient between transcript levels and cell wall components.

**Table S1. Characteristics of *GhCOBL* family genes in *Gossypium hirsutum***

| Gene name | Sequence ID | Number of Amino Acid | Molecular Weight (Da) | Theoretical pI | Subcellular localization |
| --- | --- | --- | --- | --- | --- |
| *GhCOBL1* | Ghi_A01G00256 | 289 | 32313.82 | 5.00 | Plasma membrane |
| *GhCOBL2* | Ghi_A01G03366 | 667 | 74544.17 | 9.24 | Plasma membrane |
| *GhCOBL3* | Ghi_A02G01966 | 655 | 72083.27 | 6.60 | Plasma membrane |
| *GhCOBL4* | Ghi_A03G04406 | 433 | 48416.04 | 9.13 | Plasma membrane |
| *GhCOBL5* | Ghi_A03G04411 | 442 | 49648.21 | 9.01 | Vacuole |
| *GhCOBL6* | Ghi_A06G09466 | 662 | 74276.75 | 9.10 | Vacuole |
| *GhCOBL7* | Ghi_A07G03826 | 444 | 50004.44 | 8.95 | Nucleus |
| *GhCOBL8* | Ghi_A07G10926 | 455 | 50907.45 | 9.04 | Vacuole |
| *GhCOBL9* | Ghi_A08G02926 | 430 | 48387.94 | 9.12 | Plasma membrane |
| *GhCOBL10* | Ghi_A10G10786 | 656 | 72256.94 | 5.53 | EndoPlasma reticulum |
| *GhCOBL11* | Ghi_A11G06131 | 650 | 71141.82 | 5.39 | Extracellular matrix |
| *GhCOBL12* | Ghi_A11G08886 | 380 | 42624.65 | 8.88 | Peroxisome |
| *GhCOBL13* | Ghi_A11G08891 | 433 | 49245.9 | 9.06 | Plasma membrane |
| *GhCOBL14* | Ghi_A12G01371 | 285 | 33077.01 | 6.30 | Cytoplasm |
| *GhCOBL15* | Ghi_A12G02076 | 388 | 43234.7 | 5.52 | Nucleus |
| *GhCOBL16* | Ghi_A12G10986 | 455 | 50789.29 | 8.81 | Plasma membrane |
| *GhCOBL17* | Ghi_A12G10991 | 418 | 46583.92 | 8.94 | Vacuole |
| *GhCOBL18* | Ghi_A13G01781 | 451 | 50883.43 | 6.73 | Plasma membrane |
| *GhCOBL19* | Ghi_A13G09746 | 660 | 73737.62 | 8.82 | Nucleus |
| *GhCOBL20* | Ghi_D01G00251 | 307 | 34386.2 | 4.78 | Plasma membrane |
| *GhCOBL21* | Ghi_D01G02826 | 667 | 74525.93 | 9.20 | Plasma membrane |
| *GhCOBL22* | Ghi_D02G02531 | 655 | 72035.15 | 6.60 | Plasma membrane |
| *GhCOBL23* | Ghi_D03G06061 | 520 | 57766.77 | 9.11 | Plasma membrane |
| *GhCOBL24* | Ghi_D03G06066 | 431 | 48093.19 | 8.85 | EndoPlasma reticulum |
| *GhCOBL25* | Ghi_D06G09076 | 662 | 74258.66 | 9.05 | Vacuole |
| *GhCOBL26* | Ghi_D07G03716 | 436 | 48892.22 | 8.74 | Nucleus |
| *GhCOBL27* | Ghi_D07G10291 | 455 | 51040.52 | 9.04 | Vacuole |
| *GhCOBL28* | Ghi_D08G02781 | 196 | 21543.92 | 9.26 | Vacuole |
| *GhCOBL29* | Ghi_D10G09876 | 656 | 72184.84 | 5.51 | EndoPlasma reticulum |
| *GhCOBL30* | Ghi_D11G06381 | 656 | 71772.39 | 5.14 | Extracellular matrix |
| *GhCOBL31* | Ghi_D11G08641 | 398 | 44423.74 | 8.75 | Peroxisome |
| *GhCOBL32* | Ghi_D11G08646 | 433 | 49211.89 | 9.06 | Plasma membrane |
| *GhCOBL33* | Ghi_D12G01346 | 233 | 26151.51 | 4.99 | Cytoplasm |
| *GhCOBL34* | Ghi_D12G01646 | 406 | 45801.97 | 7.15 | Nucleus |
| *GhCOBL35* | Ghi_D12G11646 | 455 | 50788.24 | 8.87 | Golgi apparatus |
| *GhCOBL36* | Ghi_D12G11651 | 109 | 12774.55 | 4.55 | Nucleus |
| *GhCOBL37* | Ghi_D12G11656 | 278 | 30857.59 | 8.85 | Nucleus |
| *GhCOBL38* | Ghi_D13G01781 | 452 | 50899.24 | 6.50 | Plasma membrane |
| *GhCOBL39* | Ghi_D13G08621 | 660 | 73631.31 | 8.68 | Chloroplast |

**Table S2. The primers used in this study**

| Primer | Sequence (5'-3') |
| --- | --- |
| *GhCOBL22*-VIGS-F | CCTGAATTCACGGTGCCAAATCCAATGC |
| *GhCOBL22*-VIGS-R | CTTGAGCTCCTTTAGAGGGGAATCCATC |
| *GhCOBL22*-qPCR-F | AAGTGGCTTGCCGTCGAATACA |
| *GhCOBL22*-qPCR-R | CACTTTGGGCTTGCGTTCTTGG |
| *GhCOBL3-F* | GCGATTGAGACTGCCGGAGATT |
| *GhCOBL3-R* | CAGGCAAGGGAACACTAGGAGC |
| *GhCOBL*10-F | CGAGGGTGGAGTGCTAGGATCA |
| *GhCOBL10-R* | TTTGAAACCCTCGGGTCGCTTT |
| *GhCOBL29-F* | TTGAAACGGCGGGGGATTTGAC |
| *GhCOBL29-R* | TAAGATCGCCACTTTGCCGAGG |
| *GhCOBL30-F* | CCGACTCATGCAACGGGATCTT |
| *GhCOBL30-R* | CGGCGTTAGAAGCAGAGACCAA |
| *GhHIS3*-F | GCGCAAAGGTTGGTGTCTTC |
| *GhHIS3*-R | TCAAGACTGATTTGCGTTTCCA |
